# Implementing cognitive behavior therapy for chronic fatigue syndrome in mental health care: a costs and outcomes analysis

**DOI:** 10.1186/1472-6963-8-175

**Published:** 2008-08-13

**Authors:** Korine Scheeres, Michel Wensing, Gijs Bleijenberg, Johan L Severens

**Affiliations:** 1 Expert Centre Chronic Fatigue, Radboud University Nijmegen Medical Centre (4628), PO Box 9101, 6500 HB, The Netherlands; 2 Centre for Quality of Care Research, Radboud University Nijmegen Medical Centre, The Netherlands; 3 Department of Health Organization, Policy and Economics, Maastricht University, The Netherlands; 4 Department of Clinical Epidemiology and MTA, University Hospital Maastricht, The Netherlands

## Abstract

**Background:**

This study investigated the costs and outcomes of implementing cognitive behavior therapy (CBT) for chronic fatigue syndrome (CFS) in a mental health center (MHC). CBT is an evidence-based treatment for CFS that was scarcely available until now. To investigate the possibilities for wider implementation, a pilot implementation project was set up.

**Method:**

Costs and effects were evaluated in a non-controlled before- and after study with an eight months time-horizon. Both the costs of performing the treatments and the costs of implementing the treatment program were included in the analysis. The implementation interventions included: informing general practitioners (GPs) and CFS patients, training therapists, and instructing the MHC employees. Given the non-controlled design, cost outcome ratios (CORs) and their acceptability curves were analyzed. Analyses were done from a health care perspective and from a societal perspective. Bootstrap analyses were performed to estimate the uncertainty around the cost and outcome results.

**Results:**

125 CFS patients were included in the study. After treatment 37% had recovered from CFS and the mean gained QALY was 0.03. Costs of patients' health care and productivity losses had decreased significantly. From the societal perspective the implementation led to cost savings and to higher health states for patients, indicating dominancy. From the health care perspective the implementation revealed overall costs of €5.320 per recovered patient, with an acceptability curve showing a 100% probability for a positive COR at a willingness to pay threshold of €6.500 per recovered patient.

**Conclusion:**

Implementing CBT for CFS in a MHC appeared to have a favorable cost outcome ratio (COR) from a societal perspective. From a health care perspective the COR depended on how much a recovered CFS patient is being valued. The strength of the evidence was limited by the non-controlled design. The outcomes of this study might facilitate health care providers when confronted with the decision whether or not to adopt CBT for CFS in their institution.

## Background

Chronic Fatigue Syndrome (CFS) is characterized by persistent or relapsing unexplained fatigue that lasts for at least six months and results in substantial reduction in previous levels of daily functioning [1]. Causes of CFS have not been found and most patients do not recover spontaneously [2]. Based on the CDC-94 criteria, CFS prevalence figures of 112 and 420 per 100.000 were found [3,4].

Cognitive behavior therapy (CBT) has proven to be an effective treatment for CFS [5,6]. Since the treatment of CFS with CBT has been available only in a few specialized university medical centers in The Netherlands, just a small minority of CFS patients can benefit from it. Nationwide implementation is needed to realize access to CBT treatment for all CFS patients. However, when decision makers have to judge whether such implementation is worthwhile and should be paid for, they need information about its costs and benefits for individual patients, the healthcare system and society.

The number of cost effectiveness analyses (CEA) of CBT for CFS and chronic fatigue (CF) are few compared to clinical evaluations. One study performed a cost consequence analysis of CBT for CF in general practice compared to regular counseling by a GP. It reported that counselling was a less costly intervention than CBT, and that both interventions led to reductions in fatigue. But no overall cost-effectiveness advantage was found for either form of therapy [7]. Another study, concerning a CEA of CBT for CF, [8] found similar cost effectiveness for CBT and graded exercise for CF. It also reported a high probability that these therapies are cost-effective compared to usual care. A third study reported a CEA of CBT for CFS and found, although with some statistical uncertainties, that regarding a time horizon of 14 months, total costs to society were lower for (ex) CFS patients that had followed CBT treatment than for those who had received usual care or guided support groups [9]. Taken together these studies indicate that CBT for CFS or CF might be cost effective for society compared to usual care.

Until now nothing is known about the costs and efficiency of implementing CBT for CFS in a clinical practice setting. It might be possible that the efficiency of CBT for CFS reduces if the implementation costs are high or if the treatment effectiveness reduces. The present study therefore evaluated the broader so-called *policy *costs and effects of a pilot implementation project in which CBT for CFS was made available in a mental health center (MHC). In a policy study all extra costs of implementing the treatment (like training therapists, informing GPs, organizing and meetings) are being included as fixed costs in the analysis, in addition to the costs and effects of just performing the treatment [10,11].

The MHC of this study was a regional middle-sized institution located in the East of The Netherlands, covering mostly rural and some urbanized areas. It had locations in four separate sub-regions and the CBT for CFS treatment was offered at two of them. This MHC was the main provider of mental health care in this area, offering outpatient and inpatient services for the full range of problems and patients.

## Methods

### Design

The evaluation was a prospective, non-controlled before and after comparison in a MHC with an observation period of 8 months.

### Implementation interventions

The implementation program contained four major interventions. First, six behavior therapists who were working in the concerning MHC were trained at the Nijmegen Expertcenter for Chronic Fatigue. They were selected on bases of their prior education in CBT and on their willingness and possibility to participate in this implementation project. None of these therapists had previous experience with CFS patients. Their number of years working as a behavioral therapist varied from two to 13 years. Second, because GPs in the region were not familiar with this new treatment setting for CFS, announcements were made in the media and information brochures were distributed to GPs. GPs could also order copies of these brochures for their waiting rooms. Third, informational interventions were performed that were directed at the patient population. These consisted of several media announcements and distribution of patient brochures. Fourth, employees of the mental health care institution were informed and, if applicable, settled into the project.

### Patients and treatment procedure

Patients who attended the treatment were all diagnosed as CFS and referred to the MHC by their GP or a medical specialist. Inclusion criteria were as follows: a GPs diagnosis of CFS (based on the CDC-94 criteria), not enrolled in a new claim for disability-related benefits, and 18 years or older. After the first session the patient had to fill in several fatigue related paper and pencil questionnaires. At 8 months follow up, when treatment was finished, the questionnaire had to be filled in again. Before starting this study it was judged by the Nijmegen Medical Hospital Ethical Commission, who indicated no need for informed consent.

To measure fatigue we used the Checklist Individual Strength (CIS20), which is a self-report measure on a 7-point Likert scale for fatigue severity over the last two weeks. The CIS has good reliability (Cronbach's alpha varying from 0,83 to 0,92) and discriminative validity [12]. Physical functioning in daily life was measured with the 'physical functioning' subscale of the SF-36 [13]. This subscale is a validated 10-item scale with a score varying from 0 (maximum of limitations) to 100 (no limitations). The Euroqol-5d was used to measure QALYs [14].

In some instances this questionnaire results contradicted the diagnosis of CFS. For example, when a psychiatric co-morbidity was found that could explain the severe fatigue. In such occasions treatment was not started and the patient was referred to another treatment program in the organization.

The CBT treatment protocol prescribes 16 sessions in a period of 8 months [15]. In this treatment, first the model of psychological and behavioral perpetuating factors of fatigue is explained to the patient. Then the patient formulates his or her goals for therapy. Afterwards the patient starts a structured graded activity program beginning with some daily minutes of walking or bicycling, which is tailored to their base line daily activity level. Subsequently, dysfunctional fatigue related cognitions are being challenged to diminish somatic attributions of fatigue, to improve a sense of control over symptoms and to facilitate behavior change. Finally a plan for work rehabilitation is outlined and worked out. Patients without a paid job focus on rehabilitation in other personal activities. The last session deals with relapse prevention and further improvement of self-control.

### Measurement and valuation

#### 1. Treatment implementation costs

Personnel costs, for therapists' trainings, coordinating activities and monthly working group assemblies, were calculated by counting the total amount of hours that concerned people had invested and by multiplying these hours with personnel's gross salary per hour, including 39% employers' charges. For training and supervision only the hours that were actually attended were calculated, per person. The hours that people had spent on the implementation were counted retrospectively by interviewing concerned people. Traveling costs related to these activities were calculated by summing up the total amount of kilometers by car and counting €0.16 per kilometer. Material costs for informing GPs and patients were determined by summing up al printing, copying and distributing costs of used materials. Accommodation costs were calculated as 10% of personnel costs [16].

#### 2. CBT integral treatment costs

For the CBT for CFS treatments integral prices were calculated, implying that all direct (executing) and indirect (overhead) costs of the MHC for offering the treatment were included in the calculation. Total costs of performed treatment sessions were determined by first summing up all therapists, diagnostic assistants and secretaries invested time per treatment. For each patient the total number of attended therapy sessions was registered. Planned sessions that were cancelled less than 24 hours before the session were also calculated. Per session one hour of work was counted for a therapist. Per treated patient 15 minutes secretary work was counted. Per intake and per post treatment session 30 minutes work for a diagnostic assistant was counted. The personnel costs for secretaries and diagnostic assistants were also based on gross salary plus39% charges. Then, for overhead costs and building use, 20% and 10% respectively of personnel costs were added to the personnel costs [16]. Treatment material costs were too small to count.

#### 3. Direct medical costs (apart from CBT treatment)

Volumes of medical consumption were measured with a paper and pencil questionnaire that was filled in by the patient at base line and after treatment. Patients were asked how many visits that they had made in the previous six months to a GP, medical specialist, physiotherapist, psychologist, psychiatrist and alternative medical practitioner. Use of home care support (average hours per week in the last 6 months) hospitalization (number of nights in 6 months) and use of (prescribed and not prescribed) medication were also asked. To value patients' medical consumption, cost prices were used as given in the Dutch cost analyses manual [16] after recalculating them to the 2004 price level (Table 1). Costs of prescribed medication were calculated based on the Dutch indicated market prices per month based on 'defined daily doses'. Six percent taxes and €6,51 pharmacy costs per client using medication were added to this market costs. Patients were asked to give a price indication per month of their costs incurred in purchasing over-the-counter medication.

**Table 1:** Cost-prices used to value the different health care volumes, measured at patient level before and after treatment.

*Health care volume*	*Cost price*
General practitioner (per visit)	€ 20.39
Medical specialist (per visit)	€ 63.99
Physiotherapist (per visit)	€ 22.96
Psychologist (per visit)	€ 125.14
Psychiatrist (per visit)	€ 88.81
Non-physician alternative medicine practitioner (per visit)	€ 48.87
Home care (per hour)	€ 21.90
Informal home support (per hour)	€ 8.38
Hospitalization (per night)	€ 333.40

#### 4. Direct non-medical costs

For each CFS patient traveling costs for attending the CFS treatment sessions were applied. Distances from patients' homes to the MHC's treatment location were found at . This distance was multiplied by each patient's total number of attended sessions. Again € 0.16 per kilometer was calculated.

#### 5. Indirect non-medical costs

Patients' lost productivity costs due to absenteeism from paid work were also measured with the paper and pencil questionnaire. The questionnaire contained questions about work and daily activities, based on the 'Health and Labour Questionnaire' [17]. The number of hours of paid work in the last two weeks was filled in. We valued the days of absenteeism from paid work with Dutch standard productivity costs specified for age, sex and education level [16] and using the human capital method. Transfer payments related to occupational disability insurances were not included since these are neither a gain nor a cost to society [10]. The productivity costs per two weeks were then multiplied by 13 to provide the costs per 6 months.

Informal care measured at baseline and after treatment with a paper and pencil questionnaire about the number of hours per week that patients had received informal care. This was costed at € 8.38 per hour [16], the wage rate for a cleaner. Time costs for patients attending the treatment sessions were excluded.

### Economic evaluation method

#### Perspective

Total costs of implementing CBT for CFS were analyzed both from a societal perspective (including also non medical costs such as travelling expenses and productivity costs, regardless of who carried them) and from a health care perspective (indicating that only medical costs were relevant) [10]. For the societal perspective we calculated costs per gained QALY. For the health care perspective instead we calculated costs per recovered patient, being a measure of health rather than a measure of general welfare, which corresponds better to the more limited scope of the health care perspective [18]

#### Calculation methods

Total costs were divided into costs for implementing the new treatment (the fixed, so called 'organizing' costs), and costs for facilitating and using the CBT for CFS treatments (the variable, so called 'executing' costs) [19]. Fixed costs were related to assembly- and organizing activities of the working group, informational interventions towards GPs and the public and training and supervising the initial therapists. Variable costs comprised of: 1. Costs for continuing the treatment facility, comprising of repeatedly providing training and supervision for new therapists (we assumed that because of personnel turnover every two years two new therapist need to be trained and supervised) and continuing PR activities; 2. Costs for clients attending the treatment sessions (e.g. traveling costs); 3. Costs for organizing and facilitating the treatments (mainly labour costs); 4. Societal costs (including use of healthcare services other than CBT and lost productivity costs due to absenteeism from paid work) and 5. Costs for performing the treatment program (time for therapist performing the treatment sessions, costs for building use, etc).

Because the time period between costs and effects was less then 12 months, we did not apply the principle of discounting. All costs were recalculated to 2004 by using the 2004 'derivative cost-of-living index figures' [20]. All cost prices included in the analyses were valued in terms of integral cost prices [16].

### Data analysis

#### Missing data

in the original database an average of 0.5 cases per item were randomly missing because some patients had failed to answer all questions of a particular questionnaire. These missing data were filled in with the median value for the particular item. In the cases of missing data due to loss of follow up the method of last observation carried forward was used [21], indicating that intake measurements were used as post treatment. Analyses were performed on basis of intention to treat; patients who attended an intake but did not start treatment and patients who dropped out of treatment were all included in the cost analyses.

#### Cost and outcome calculations

Given the non-controlled design of the present study, it did not fulfil the criteria of a 'full economic evaluation' [10], and hence the usually calculated incremental costs effectiveness ratios (ICER) could not be analysed. In stead we calculated cost outcome ratios (CORs). Cost outcome ratios (COR) are concerned with the joint difference in costs and outcomes before and after (implementing and) performing a certain treatment [10]. This ratio thus indicates the financial investment that is needed to gain a certain treatment effect, based on the assumption that autonomous change regarding the patients is negligible.

The COR was calculated in two ways. First by defining treatment effect as 'percentage of recovered patients' (health care perspective), and second by using quality adjusted life-years (QALYs) as a measure for treatment effect (societal perspective). The recovery rate was analysed by calculating the percentage of patients experiencing significant clinical improvement (CSI). Patients were defined as being CSI at post treatment if they had a reliable change index > 1.96 on the CIS fatigue severity subscale [22], a fatigue severity score <= 35 and a Rand-36 physical functioning score > = 65 [12]. Quality of life was measured using utility scores of the Euroqol [14]. This utility score, lying between 0 (health state equal to death) and 1 (perfect health state), represents the QALY due to some intervention.

Since the health care costs were measured over a period of 6 months, while the individual durations of treatment differed between 2.2 and 16.2 months, all medical and non-medical costs at follow up were extrapolated to the individually defined treatment period, before including them in the cost outcome analyses.

Utility scores were measured two times, at intake and at follow up. Since the difference in utility scores between the two measurements was presumably reached gradually instead of at once, and because the duration of treatment differed per patient, the gained QALYs at post treatment were calculated as: 0.5 * (utility score post treatment – utility score intake)/12 * individual number of months of treatment [10].

#### Analysis of uncertainty

Because it was presumed that, as usually, the measured medical costs would follow a skewed distribution, a normality assumption would be problematic when estimating confidence intervals. Therefore the non-parametric bootstrap method [23] was used to quantify the uncertainty of the calculated COR. In the bootstrap method this uncertainty is quantified by plotting cost-effectiveness acceptability (CEA) curves by means of repeated re-sampling of the costs and outcome data (the bootstrapping), which generates a distribution of mean costs and outcomes of two situations [24]. These distributions are then used to calculate the probability that one of the situations is the optimal choice, given a range of possible maximum values (ceiling ratio) that a decision maker might be willing to pay for a unit of improvement in outcome. Because the present study did not calculate cost effectiveness, we used the term 'COR acceptability curve' instead of 'ICER acceptability curve'.

#### Scenario calculation

For both the societal and the healthcare perspective, the COR of implementing CBT for CFS in a MHC was also calculated for a period of 5 years.

## Results

### Descriptives

**Figure 1:**
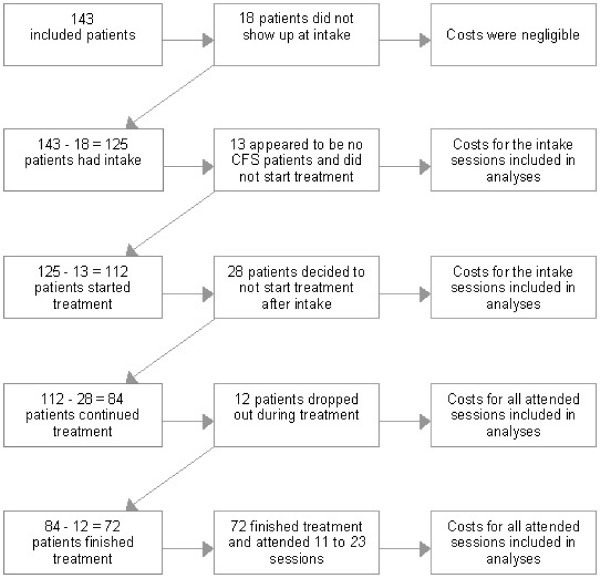
Patient flow.

Figure 1 presents the patient flow. From the 143 patients that entered the MHC during the observation period, 18 'no show' patients never showed up at the intake session. Since they only caused negligible costs they were excluded from this study. The remaining 125 patients were included. At intake 13 patients appeared not to fulfil the diagnostic criteria for CFS, these patients did not start treatment. Of the 112 patients that started treatment, 28 dropped out of treatment quickly after the intake session. Of the 84 patients that followed treatment 12 dropped out half way or later and 72 finished treatment.

### Missing data

At 8 months follow up 74 of the 84 treated patients filled in the questionnaire and 10 patients failed to do this (7 drop out patients and 3 treatment completers). Of the 13 'non CFS' and the 28 'non starting' patients their intake measurements were used as post treatment, since no treatment effect was to be expected within less than 2 sessions.

### Sample characteristics

Patients' characteristics are shown in Table 2. Of the 77 patients (62%) that had a paid job (42 fulltime and 35 part time) 54 were actually working and 23 were on sickness benefit.

**Table 2:** Patients' characteristics (N = 125)

Categorical variables	(N/%)
Sex (man/women)	42 (34%)/83 (66%)
Higher education	51 (41%)
Having a paid job	77 (62%)
Married/living together/living with parents	98 (78%)
	
Continuos variables	M (SD)
Age	38.7 (10.2)
Duration of fatigue (years)	6.3 (7.0)
Fatigue severity (Cis20)	48.3 (8.0)
Physical impairment (Rand 36)	54.0 (23.4)
Social impairment (Rand 36)	41.5 (23.7)
Psychosocial well-being (SCL-90)	165.1 (42.1)

### Treatment characteristics

The mean duration of the 84 performed treatments was 8.4 months (SD 3.3) and varied from 2.2 to 16.2 months. No relations were found between duration of treatment and several other variables, like treatment effect, decrease in medical consumption or lost productivity costs after treatment. The mean number of treatment sessions was 14.5 (SD 5.6) and varied from 6 to 23 sessions.

### Treatment effects

Effect based on fatigue severity: after treatment 46 of 125 patients (37%) were recovered. Effect based on Euroqol: the mean utility score at intake was 0.57 (SD 0.27) and post treatment 0.65 (SD 0.30) (table 3).

**Table 3:** Mean utility scores at intake and 8 months follow up (N = 125)

	*Intake*	*Follow up*	*Δ follow up – intake*	*95% CI*	*P*
Mean (SD)	0.57 (0.27)	0.65 (0.30)	0.078 (0.028)	0.03 to 0.09	< 0.001

### Costs results

Table 4 shows the total fixed and variable costs of preparing and introducing the implementation of CBT for CFS, with a total of € 90.765 and € 59.300 respectively. The costs results of performing and using CBT (table 5) reveal that per patient a mean of € 597 were spent per CBT treatment.

**Table 4:** Costs of developing and introducing the implementation of CBT for CFS divided in fixed and variable costs.

	*Volume*	*Calculated costs value per volume*	*Costs*
Personnel costs			
Fixed			
Therapists	647 hours	€ 55.50/hour	€ 35.909
Management	312 hours	€ 73.17/hour	€ 22.829
Others	793 hours	€ 35.87/hour	€ 28.444
Total fixed			€ 87.182
Variable			
Therapists	460 hours	€ 55.50/hour	€ 25.530
Management	20 hours	€ 73.17/hour	€ 1.463
Supervisor	266 hours	€ 62.52/hour	€ 16.625
Others	6 hours	€ 35.87/hour	€ 215
Total variable			€ 43.833
TOTAL personnel costs			€ 131.015
			
*Material costs*			
Fixed			
Building use	10% of personal costs		€ 8.718
PR activities	2000 information letters and brochures		€ 1.705
Total fixed			€ 10.423
Variable			
Building use	10% of personal costs		€ 4383
PR activities	2500 information letters and brochures		€ 1.983
Total variable			€ 6.366
TOTAL material costs			€ 16.789
			
Traveling costs			
Fixed			
Therapists	3605 km	€ 0.18/km	€ 649
Management	3328 km	€ 0.18/km	€ 599
Others	1411 km	€ 0.18/km	€ 254
Total fixed			€ 1.502
Variable			
Supervisor	5300 km	€ 0.18/km	€ 954
Therapists	1275 km	€ 0.18/km	€ 230
Management	40 km	€ 0.18/km	€ 7
Total variable			€ 1.191
TOTAL traveling costs			€ 2.693
			
TOTAL fixed costs			€ 90.675
TOTAL variable costs			€ 59.300
TOTAL 'developing and introducing' costs			€ 149.975

All volumes were valued in terms of Dutch integral cost prices at the price level of 2004 (Oostenbrink et al., 2004).

**Table 5:** Mean costs per patient (in €) of using and performing CBT for CFS in mental health care (N = 125)

	*Mean*	*SD*	*Median*	*Max*
Personal costs of CBT treatment (only therapist costs)	€ 417	€ 314	€ 435	€ 1349
Personal costs of CBT treatment (secretary and test assistants costs)	€ 28	€ 7	€ 34	€ 34
Overhead costs and costs for building facilities	€ 143	€ 101	€ 146	€ 442
Patients travelling costs (return price)	€ 9	€ 6	€ 7	€ 43
TOTAL mean costs per patient of using and performing CBT for CFS	€ 597	€ 424	€ 628	€ 17892

Table 6 gives the amounts of medical care other than CBT treatment. These results were used for calculating all (non) medical costs (table 7). As can be seen, total medical costs decreased from € 1.112 per six months before treatment to € 810 after treatment (95% CI -€ 784 to -€ 26). Total non-medical costs also decreased, from € 1.249 per six months before treatment to € 1.012 after treatment (95% CI -€ 813 to € 271).

**Table 6:** Volumes of medical consumption (except CGT for CFS treatment) over a period of 6 months measured at patients level at intake and follow up (N = 125).

	Intake			Follow up		
	N	*Mean (SD)*	*Median*	N	*Mean (SD)*	*Median*

*Medical care*						
GP (number of visits)	111	3.1 (3.7)	2	93	2.0 (1.9)	2
Medical specialist (n.o. visits)	55	1.2 (1.8)	0	38	0.9 (1.5)	0
Physiotherapist (n.o. visits)	29	3.8 (9.0)	0	23	2.7 (7.4)	0
Psychologist, other than CBT for CFS	22	1.0 (3.1)	0	13	0.4 (1.4)	0
Psychiatrist (number of visits)	9	0.2 (1.2)	0	7	0.2 (1.2)	0
Home care (hours per 6 months)	21	26.9 (104.3)	0	23	22.7 (88.4)	0
Hospitalisation (nights)	13	0.4 (2.0)	0	9	0.2 (1.5)	0
Prescribed medication (yes/no)	92	77%		87	72%	
*Non-medical care*						
Informal home care (hrs per 6 months)	37	132.3 (268.3)	0	33	110.6 (182.8)	0
Altern. med. practitioner (n.o. visits)	30	1.0 (2.3)	0	21	0.8 (2.0)	0
Non prescribed medication (yes/no)	56	53%		27	35%	

N = the number of patients that had been using this form of healthcare

**Table 7:** Mean medical and non-medical costs per 6 months measured at patient level at intake and follow up (N = 125).

	Intake			Follow up		
	*Mean*	*SD*	*Median*	*Mean*	*SD*	*Median*

Medical costs						
GP	€ 63	€ 67	€ 41	€ 41	€ 40	€ 41
Medical specialist	€ 77	€ 103	€ 0	€ 58	€ 95	€ 0
Physiotherapist	€ 87	€ 202	€ 0	€ 62	€ 181	€ 0
Psychologist	€ 125	€ 377	€ 0	€ 50	€ 172	€ 0
Psychiatrist	€ 18	€ 107	€ 0	€ 18	€ 95	€ 0
Home care	€ 589	€ 1629	€ 0	€ 498	€ 1235	€ 0
Hospitalisation	€ 134	€ 720	€ 0	€ 67	€ 509	€ 0
Prescribed medicine	€ 19	€ 50	€ 3	€ 16	€ 52	€ 3
						
*Non medical costs*						
Alternative med. pr.	€ 56	€ 114	€ 0	€ 39	€ 94	€ 0
Non prescr. medicine	€ 52	€ 121	€ 0	€ 25	€ 53	€ 0
Informal homecare	€ 1109	€ 2322	€ 0	€ 927	€ 1573	€ 0
Travelling costs	€ 32	€ 32	€ 23	€ 21	€ 28	€ 11

Total medical costs	€ 1112	€ 2258	€ 362	€ 810	€ 1350	€ 241
Total non-medical costs	€ 1249	€ 2396	€ 112	€ 1012	€ 1822	€ 72

In table 8 the figures on work and absenteeism are given, showing that the mean number of working hours according to contract had fallen from 16.4 per week before treatment to 14.9 after treatment (95% CI -5.4 to 3.2). The number of real worked hours however had risen from 9.3 before treatment to 11.4 hours per week after treatment (95% CI – 2.6 to 5.5), implying that the number of lost productivity hours and its costs decreased, from € 218 per patient per week before treatment tot € 122 after treatment (95% CI -€ 173 to -€ 6).

**Table 8:** Patient volumes of work and lost productivity costs per week, measured at patient level before and after treatment (N = 125).

	Intake				Follow up			
	*Mean*	*SD*	*Median*	*Max*	*Mean*	*SD*	*Median*	*Max*

Number of contract hours	16.2	16.3	10	40	14.9	16.2	7	40
Number of worked hours	9.4	13.5	0	45	11.4	14.7	0	46
Absenteeism in hours	7.4	12.3	0	40	4.1	8.8	0	40
Lost productivity costs	€ 218	€ 392	€ 0	€ 1544	€ 122	€ 292	€ 0	€ 1544

### Cost outcome ratios

**Figure 2:**
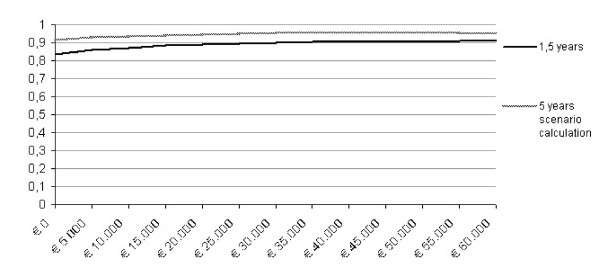
**Acceptability curve showing the probability that implementing CBT for CFS has a favorable cost outcome ratio over a range of willingness to pay regarding societal costs per QALY**. Societal willingness to pay for a CFS patient's gained QALY.

From the societal perspective the mean societal costs per patient per six months were € 8.030 before implementing CBT for CFS and € 6.869 after it (95% CI -€ 3.489 to € 1.083). The mean gained QALY per patient was 0,03. Given the lower cost level and a higher health state of patients, the COR-estimate indicates dominancy. The five years scenario calculation analysis, in which the total amount of treated patients was up-scaled to 3.33 times the amount of patients that were treated in the implementation period of 1,5 years (also figure 2), revealed a greater than 90% probability for a favorable COR for all acceptability thresholds.

**Figure 3:**
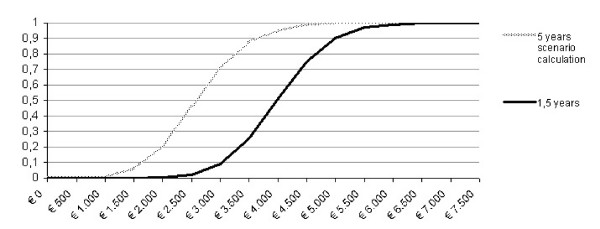
**Acceptability curve showing the probability that implementing CBT for CFS has a favorable cost outcome ratio over a range of willingness to pay regarding health care costs per recovered patient**. Willingness to pay for recovering a CFS patient.

From the health care perspective it was found that mean costs per patient per six months were € 1.117 before implementation and treatment and € 2.586 after it (95% CI € 958 to € 1.876). Given the recovery rate of 37% the COR of implementing CBT for CFS was € 5.320 per recovered CFS patient. The COR acceptability curve (figure 3) shows that the probability that implementing CBT for CFS has a favorable COR is 100% when the decision maker values a recovered CFS patient at least € 6.500.

The 5 years scenario calculation (also figure 3) showed that the 100% guarantee for an acceptable COR was reached at the willingness to pay threshold of € 4.500.

### Sensitivity analysis

In sensitivity analysis applied for the societal perspective, the costs for (informal) home care and productivity costs were varied. As also has been found in other studies [7,8] (informal) home care appeared to cause major costs. Besides that questions may be raised about the accuracy of the measured amounts of home care. It is a difficult aspect to measure, for example the distinction between informal care and normal household activities is not clearly defined, both for researchers and for patients, especially if the informal caregiver shares a household with the patient [25]. Since patients have a tendency to overestimate their hours of informal care, we performed a calculation reducing informal home care to 50% and leaving it out at all. In a third calculation both informal and formal home care were omitted from the analyses. These calculations showed that if informal home care was omitted from the analysis, and when both informal and formal homecare were omitted, the probability that implementing CBT for CFS has an acceptable COR remained above 80% for all acceptability thresholds.

In addition, two extra analyses were done, in which productivity costs were set to 70% and to 30% of the original base case level. This revealed a drop in cost savings of CBT to -€ 16.800 and -€18.730 respectively. It appeared that implementing CBT for CFS remained dominant at both the 70% and the 30% level.

Finally, to get an impression of this study's results when compensating for spontaneous recovery, an additional analysis was performed. This was done from the health care perspective, presuming a spontaneous recovering rate of 5% [2], implying a recovery rate due to treatment of 32%. It revealed that the COR would rise from € 5.320 to about € 5.969 per recovered patient.

## Discussion

This study has shown that from a societal perspective the cost outcome ratio (COR) after implementing CBT for CFS in a MHC was dominant compared to before. From a healthcare perspective the COR after implementation was more costly but also more effective than before, and the 100% probability that the COR is acceptable was reached at the willingness to pay threshold of € 4.500 is positive. Given that CBT is the only effective treatment for CFS and has been scarcely available until now, this is relevant information in favor of nationwide implementation. Although some studies have already examined the cost effectiveness of behavioral treatments for chronic fatigue (CF) [7,8] and for CFS [9], there has been no research into the cost effectiveness of such a treatment that also took into account the costs of designing the implementation interventions needed for implementing the treatment and the costs of actually implementing the treatment in a non-academic setting. Such a study implies a less homogenous patient population and less control over the content of performed treatment sessions than an academic setting can guarantee.

Concerning age and gender, the patient population was fully representative of the CFS population. Compared to other trials in the area of CFS, the baseline fatigue severity was a little lower and relatively many patients had a paid job [26,27]. These differences could be explained by the fact that the treatment facility at the mental health care institution was more easily accessible. Patients may be recognized as CFS by their GP and referred to CBT in an earlier phase than patients referred (mostly by a medical specialist) to a specialized hospital setting.

As was also found in earlier cost effectiveness studies, [8,9] an overall lower use of health care facilities was measured after CBT for CFS than before it. This may be explained by the fact that during treatment with CBT patients are instructed not to use other treatments or medication and by the fact that when starting treatment all patients were diagnosed as CFS. Looking for a diagnosis and a lack of affective treatment are the main reasons for CFS patients' high use of health care facilities [28]. Concerning work productivity, fewer patients had a paid job after treatment than before, but the mean hours of paid work per week had increased after treatment. Given the short time horizon (8 months) the full influence of CBT for CFS on work productivity might be revealed to be larger and the impact on cost-effectiveness more pronounced.

In this study we used a conservative method, last observation carried forward, in cases of missing data. This imputation method might have influenced the results in a conservative, negative direction. However the proportion of missing data was in our opinion rather small (< 12%) thus the chance that significantly different results were obtained is small.

A serious limitation of this study is it's non-controlled before and after design, which implies that incremental cost effectiveness compared to a natural course control group, or compared to a guided support group controlling for any placebo effect, could not be analysed. However, the incremental cost effectiveness ratio (ICER) of CBT for CFS compared to usual care was recently reported by Severens et al. [9]. The focus and contribution of the present study was primarily to investigate costs and consequences of implementing this evidence based treatment in a clinical practice setting. This is a relevant issue in bridging the gap between science and research, since proven (cost) effectiveness under laboratory conditions of RCTs does not guarantee the same in the practice field of health care. Both smaller treatment effects due to the less controlled situation and accompanying costs of including costs for implementing the treatment might change the cost-outcome ratio.

Another weak point in this study is the variable follow up time. Although the mean time period between intake and post treatment was 8.4 months, and analyses were done using this time horizon for all patients, the real time interval varied considerable. The problem hereby is that in fact we do not know what this implies for the results that were found.

A strong point though is the fact that, besides the usual included medical-, productivity-, and patient related costs also protocol driven- and implementation related costs were included [29], giving a more complete and more relevant view on the cost and outcomes of providing nationwide CBT for CFS.

## Conclusion

To conclude, the results of this study suggest that implementing cognitive behavioral therapy for chronic fatigue syndrome in a mental health center is feasible and advisable. This strategy appeared to be dominant (resulting in lower costs and higher health states) compared to the starting situation from a societal perspective. From a health care perspective the implementation also implied better health states, but also higher costs, and the probability of a positive cost outcome ratio depended on how much value is placed on a recovered CFS patient. The outcomes of this study might facilitate the decision for health care providers whether or not to adopt CBT for CFS in their institution.

## Competing interests

The authors declare that they have no competing interests.

## Authors' contributions

KS collected all data, performed the statistical analysis together with JLS and wrote manuscript. JLS contributed to the development of the study design concentrating on the costs aspects, advised about the performance of the statistical analysis, checked the analysis and results and provided the Bootstrap program. MW contributed to the development of the study design concentrating on the implementation outcome aspects and revised the manuscript critically several times. GB delivered the treatment outcome measurement scales, helped with interpreting the results and helped to draft the manuscript. All authors read and approved the final manuscript.

## Pre-publication history

The pre-publication history for this paper can be accessed here:


